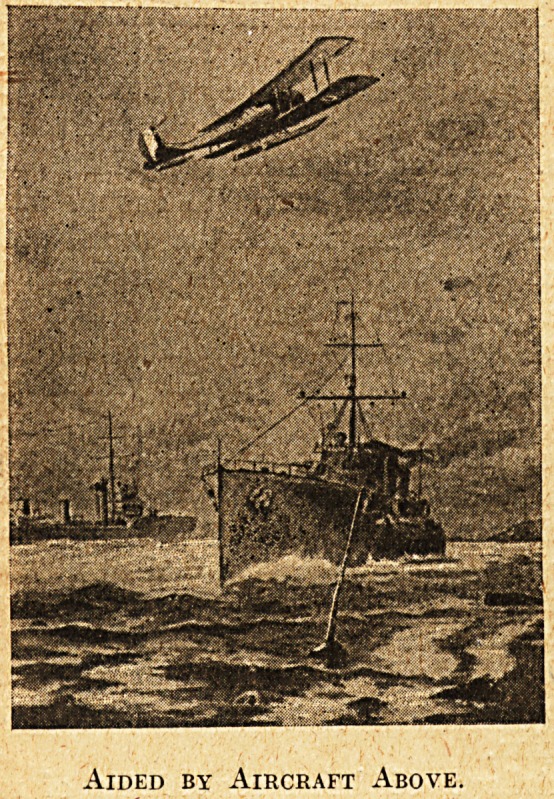# At the Ocean Gates of Britain

**Published:** 1919-05-17

**Authors:** 


### NOTES OF A NAVAL SURGEON.

### III.—At the Ocean Gates of Britain.

Crowded in the shelter of a giant bastion of the north-west Irish coast, protected from the screaming gales of the Atlantic, is found a collection of iron huts, blotched cunningly with the elemental colours of the landscape and clinging to that desolate,

The Dash of the Nearest Destroyer.

rockbound coast like a limpet growth of civilisation. Paths are cut and cindered in the yellow peat grass, whitened stones mark the angles, not for beauty but to direct him who must venture into the black darkness of night amid those crags. A tolling bell marks out the "watches," a figure in blue or glistening waterproof may momentarily appear, and above, towering beyond a ragged white ensign, are the giant limbs and network of a wireless aerial. Such is this naval outpost; tiny, seemingly at the mercy of Nature's whim, but to England of vital worth. Through those singing wires has been controlled one-quarter of the food that reached these islands at the time of our greatest need.

Westward, beyond five hundred miles of long, green rollers, now bathed in the light of a fitful winter sun, is slowly approaching a British warship, thrusting a high, sharp bow through the Atlantic swell, turning with signal flying at the foremast to round up some straggler from her thirty-ship convoy, then gliding once more to her station ahead as the whole fleet, with the mathematical accuracy of geese on the wing, pursue the zig-zag course she sets them. Her wireless talks at will with the station perched on the cliffs, and so with the Admiralty, where officers, to whose skill sufficient praise can never be given, guide her movements through a maze of dangers and the greatest game of chess the world has ever known.

The doctor is an insignificant atom in all these issues. The mere mention of his presence calls almost for an apology, but he takes his turn in forging a tiny link in the chain. Below decks in the warship, within a cramped, steel-walled cabin, lighted by a single electric globe and equipped with wooden table and chair of that simplicity and massiveness which only a naval carpenter can achieve, for four hours at a stretch sits "Doc" in the capacity of cypher officer. Head and shoulders alone are visible; the rest is enclosed in a large ship's sack, for warmth and not agility is the major consideration. Behind him in a great iron box, each in its separate leaden cover (to ensure sinking if the ship be lost), are locked the secret codes and cyphers of the Navy. The steady roll and hum of the vessel, the light's cold gleam, and the still, enwrapped figure amply suggest the waiting if not the watching aspect of the popularly ascribed naval motto.

Minutes glide by with only the lurch of a change in the ship's course to break the dead monotony until, suddenly, a fluttering paper, covered with row after row of cyphers, is thrust in at the door. "Doc" wriggles from his sack, gropes in the precious box, and makes all speed to translate those figures into words. The little station on the cliffs has spoken to H.M.S. —— a timely word of warning and direction.

The privilege of being a spectator of repeated gauntlet-running by food and troop convoys coming from the west compensated for many "moans" which a landsman medico might otherwise acquire. Apart from the beauty of all ships at sea—and they have a unique power of fascination—there was always enthralling uncertainty as the little fleets drew nearer and nearer to the danger zones of our narrow home waters.

Her Thirty-ship Convoy.

☛ Previous articles appeared March 22 and April 19, page 59.

**Notes of a Naval Surgeon**—(*continued*).

Two or three hundred miles out rendezvous was made with escort destroyers, strung out abreast, miles apart according to visibility and half buried in the heaving seas. Falling into station on the convoy's outskirts, and ever hunting untiringly, these magnificently handled vessels have done far more service and deserved more praise than has ever been publicly realised. Often one saw them, beaten by gales, put temporarily about, only to turn and drive once more into the fierce head-winds the moment the faintest chance occurred; trying again

AIDED BY AIRCRAFT ABOVE.

and again to reach, before they arrived in the submarines' hunting-grounds, a group of great helpless merchantmen rolling comfortably eastward with the weather astern.

Slow ships and stragglers were frequently caught by the enemy and great efforts were made by the escorts to keep such vessels in station. Arguments and the delinquents' protests conducted with flag, siren, or wireless had invariably the same ending. In response a speck on the horizon astern would become a great black smoke smudge as her stokers desperately toiled, driving the squat hull of the tramp half a knot faster than ever before, until morning would find her rusty bows again rising and falling in their appointed place in the fleet.

Seldom, if ever, did a convoy enter the narrow North Channel or the lower Irish Sea without a submarine lurking in its vicinity and attempting to attack. The momentary feather of a periscope, the white wake of a long-range torpedo, the lightening dash of the nearest destroyer, the dull boom of her depth-charges, and then, with the convoy keeping unswerving course, the escort vessels would circle to the threatened quarter and, aided often by aircraft above, harry and chase the enemy like gulls with a new-found feeding, dwindling astern as the supply-ships of England bore steadily on their way.

The war at sea has presented many wonderful pictures, and the M.O., with his relative freedom in time and movement had exceptional opportunities to appreciate them. His cypher duties kept him in touch with an enormous amount of sea activity for many miles around his own ship, whose wireless intercepted all messages of appropriate wave-lengths. Admiralty patrol vessels and stations kept up a magnificently organised series of signals giving every located submarine movement, and it was often possible to sit below in the cypher-room and trace with a chart the approximate daily excursions of many a "Fritz," who probably thought himself quite unobserved.

Of medical work, one fears there has been no mention, but that met with on convoy work in no way differs from common experiences of the high seas. The rare circumstance of transferring an M.O. in mid-ocean to another ship in which acute illness has occurred, is only possible in the finest weather. Even then, to the landsman, it is not an enjoyable proceeding. The author experienced one such visit to an American destroyer so hard hit with influenza that she was soon forced to return to base—and one can at least say that the affair demonstrated the polished seamanship, in handling both large vessels and small boats, that the British sailor can display.

## Figures and Tables

**Figure f1:**
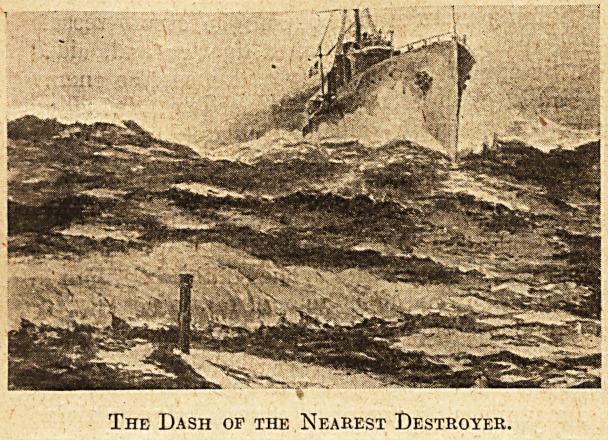


**Figure f2:**
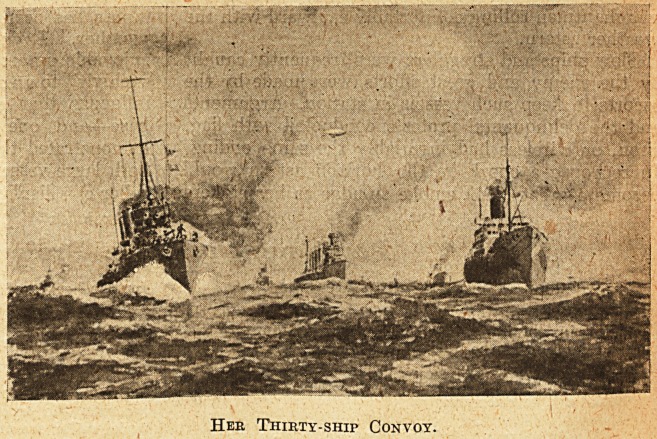


**Figure f3:**